# Constructing and validating a predictive nomogram for osteoporosis risk among Chinese single-center male population using the systemic immune-inflammation index

**DOI:** 10.1038/s41598-024-63193-7

**Published:** 2024-06-02

**Authors:** Hang Zhuo, Zelin Zhou, Xingda Chen, Zefeng Song, Qi Shang, Hongwei Huang, Yun Xiao, Xiaowen Wang, Honglin Chen, Xianwei Yan, Peng Zhang, Yan Gong, Huiwen Liu, Yu Liu, Zixian Wu, De Liang, Hui Ren, Xiaobing Jiang

**Affiliations:** 1 The First Clinical Medical College, Guangzhou University of Chinese Medicine, Guangzhou, 510405 China; 2 Medical Department, Dalian University of Technology, Dalian, 116024 China; 3 The Third Clinical Medical College, Guangzhou University of Chinese Medicine, Guangzhou, 510405 Guangdong China; 4 The First Affiliated Hospital of Guangzhou University of Traditional Chinese Medicine, Guangzhou, 510405 China; 5 The Spine Surgery Department, Second Affiliated Hospital of Guangzhou Medical University, 250 Changgang East Road, Haizhu District, Guangzhou, 510260 Guangdong China

**Keywords:** Nomogram, Osteoporosis, Male, Validation, Metabolic bone disease, Inflammation, Endocrine system and metabolic diseases

## Abstract

Osteoporosis (OP) is a bone metabolism disease that is associated with inflammatory pathological mechanism. Nonetheless, rare studies have investigated the diagnostic effectiveness of immune-inflammation index in the male population. Therefore, it is interesting to achieve early diagnosis of OP in male population based on the inflammatory makers from blood routine examination. We developed a prediction model based on a training dataset of 826 Chinese male patients through a retrospective study, and the data was collected from January 2022 to May 2023. All participants underwent the dual-energy X-ray absorptiometry (DXEA) and blood routine examination. Inflammatory markers such as systemic immune-inflammation index (SII) and platelet-to-lymphocyte ratio (PLR) was calculated and recorded. We utilized the least absolute shrinkage and selection operator (LASSO) regression model to optimize feature selection. Multivariable logistic regression analysis was applied to construct a predicting model incorporating the feature selected in the LASSO model. This predictive model was displayed as a nomogram. Receiver operating characteristic (ROC) curve, C-index, calibration curve, and clinical decision curve analysis (DCA) to evaluate model performance. Internal validation was test by the bootstrapping method. This study was approved by the Ethic Committee of the First Affiliated Hospital of Guangzhou University of Traditional Chinese Medicine (Ethic No. JY2023012) and conducted in accordance with the relevant guidelines and regulations. The predictive factors included in the prediction model were age, BMI, cardiovascular diseases, cerebrovascular diseases, neuropathy, thyroid diseases, fracture history, SII, PLR, C-reactive protein (CRP). The model displayed well discrimination with a *C*-index of 0.822 (95% confidence interval: 0.798–0.846) and good calibration. Internal validation showed a high C-index value of 0.805. Decision curve analysis (DCA) showed that when the threshold probability was between 3 and 76%, the nomogram had a good clinical value. This nomogram can effectively predict the incidence of OP in male population based on SII and PLR, which would help clinicians rapidly and conveniently diagnose OP with men in the future.

## Introduction

Osteoporosis (OP) is a prevalent metabolic disease characterized by bone mass loss and deterioration of bone microstructure^1^. It can lead to bone fragility, increased fracture risk and mortality^2^. While more common in females, men also experience this condition^3^. Unlike OP in postmenopausal women caused by hormonal changes, OP in males is primarily due to various age-related factors. China, with one of the largest populations in the world, is now facing the challenge of an aging population^4^. From 2008 to 2015, the prevalence of OP in China gradually increased from 14.94 to 27.96% in 2015^5^. According to an epidemiological survey conducted by Chinese Medical Association Osteoporosis and Bone Mineral Salt Branch in cooperation with the Chinese Center for Disease Control and Prevention (CDC) revealed that 46.9% of the male population in China had low bone mass and required prevention and treatment^6^. The risk of fragility fracture generally increases with age. While women have a higher risk of fragility fractures, in the male population, fragility fractures are associated with a higher mortality rate^3^. Therefore, men with OP can have more serious consequences. Unfortunately, the current focus on OP in men still insufficient, so the level of diagnosis and prevention of OP in men needs to be improved compared to women^7^. Regular diagnostic method for OP is the dual-energy X-ray absorptiometry (DXEA) and assessed by bone mineral density (BMI)^8^. However, first of all, the rural population in China represents a large proportion of the total population^9^. Considering the economic status of many rural elderly populations, they have difficulty accessing relatively expensive medical diagnostic technique^10^. Moreover, many men with OP are unaware of their condition and do not undergo DXA examinations until they experience symptoms like bone pain or fractures. This underscores the importance of early diagnosis and prevention of OP in clinical settings.

Prior research has identified systemic inflammation markers like the systemic immune-inflammation index (SII), neutrophil-to-lymphocyte ratio (NLR), lymphocyte-to-monocyte ratio (LMR), and platelet-to-lymphocyte ratio (PLR) as novel diagnostic biomarkers for predicting OP risk in women^11^. These inflammatory biomarker indices can be calculated from routine blood tests, making them accessible, cost-effective and convenient. Notably, there is no current assessment tool for OP screening based on systemic inflammatory markers in male population. Therefore, our study aimed to develop a practical nomogram utilizing systemic inflammatory markers and patient-related clinical data to aid physicians in promptly and accurately diagnosing OP in men.

## Methods

### Participants

Research approval was obtained from the First Affiliated Hospital of Guangzhou University of Chinese Medicine’s Ethics Committee and conducted in accordance with the relevant guidelines and regulations. From January 2022 to May 2023, we selected 826 male patients aged 40 and above in the First Affiliated Hospital of Guangzhou University of Chinese Medicine. Patients who had characteristics as follows were excluded: (1) patients who were younger than 40 years old; (2) patients who had the blood test within a week after surgery; (3) patients who are undergoing acute infections; and (4) patients who had missing data regarding clinical examinations. Eventually, 826 male patients were included in the present study and all participants were fully informed of this retrospective study. Data such as age, body mass index (BMI), and disease of patients were collected from medical records.

### Clinical examination

All participants enrolled in this study underwent the dual energy X-ray Absorptiometry (DEXA) scanning (Wi model, HOLOGIC Inc.). Measuring part including the lumbar vertebral bone 1–4, left femoral neck and left hip joint. The accessed bone mineral density (BMD) values were then transformed into *T*-score based on corresponding coefficient. According to the OP diagnosis criteria from the World Health Organization (WHO)^12^, the definition of OP is *T*-score ≤ − 2.5, and the definition of osteopenia is − 2.5 < *T*-score < − 1. In current study, participants with *T*-score > − 2.5 were divided into non-OP group, and others were divided into OP group.

Blood routine examination required fasting 8–12 h before blood drawing, and venous blood was collected from 8:00 a.m. to 9:00 a.m. for testing. Then, the blood sample were sent to clinical laboratory and analyzed by using automatic blood cell analyzer. Various blood parameters such as neutrophil counts, lymphocyte counts, monocyte counts, platelet counts, erythrocyte sedimentation rate (ESR), white blood cell count (WBC) and C-reactive protein (CRP) were measured and recorded. The calculation method for inflammatory markers were consistent with the literature^13^: The SII was calculated as the platelet count × neutrophil count/lymphocyte count. The NLR was calculated as the neutrophil count/lymphocyte count. The LMR was calculated by dividing the lymphocyte count by monocyte count. The PLR was calculated by dividing the platelet count by the lymphocyte count.

### Statistical analysis

Statistical analysis was performed through the SPSS software (version 25.0, IBM Inc.) and R software (version 4.2.0; http://www.R-project.org). Categorical variables were shown as a percentage, and the significance was determined using *χ*^2^ or Fisher’s exact test. The value of *P* < 0.05 (two-sided) was considered statistically significant. The quantitative variables were converted to categorical variables based on cut-off values. In order to construct the predictive model, the least absolute shrinkage and selection operator (LASSO) method was used to filter characteristic variables with nonzero coefficients^14^. After that, multivariable logistic regression analysis was used to build a predicting model by incorporating the variables selected by the LASSO regression model. These characteristic variables in the multivariable logistic regression model were represented by a regressive coefficient (β), odds ratio (OR) with 95% CI, and *P*-value. The statistical significance levels were all two sided.

Calibration curves were plotted to assess the calibration of the risk-predicted nomogram. A significant Hosmer–Lemeshow test statistic (*P* < 0.05) indicated that the model did not calibrate well, suggesting a potential discrepancy between predicted probabilities and observed outcomes, which could impact the model's effectiveness^15^. In order to quantify the discrimination of the risk-predicted nomogram. *C*-index was calculated. The area under the receiver operating characteristic (ROC) curve (AUC) can comprehensively assess performances across the risk spectrum^16^. Bootstrapping validation (1,000 bootstrap resamples) was performed to calculate a corrected *C*-index^17^. Decision curve analysis (DCA) was used to evaluate the clinical usefulness of the risk-predicted nomogram by quantifying the net benefits at different threshold probabilities in the male patient cohort^18^. The calculation method of net benefit is to subtract the proportion of true positive patients from the proportion of all false positive patients, and balance the relative harm of giving up intervention and the negative consequences of unnecessary intervention^19^.

### Ethical approval

This study has been approved by the ethic committee of the First Affiliated Hospital of Guangzhou University of Chinese Medicine (JY2023012). All authors of this study confirm that they have obtained the informed consent of all subjects and/or their legal guardians.

## Results

### Patients’ characteristics

After screening, a total of 826 male patients involved in our study. Among them, a total of 140 patients were defined as OP, and other 686 patients were defined as non-OP according to the diagnosis criteria. All patients have completed relevant examination. The cohort consisted of 165 patients with hypertension, 79 patients with diabetes, 92 patients with fracture history, 101 patients with cardiovascular disease, 49 patients with cerebrovascular disease, 27 patients with tumor, 18 patients with chronic renal failure, 17 patients with thyroid disease, 6 patients with rheumatism, 30 patients with neuropathy. All data of patients including demographic and disease features in the two group are shown in Table 1.

**Table 1:** Differences between demographic characteristics of the male patients. Categorical parameters were shown as percentage.

Parameters	Non-OP (n = 686)	OP (n = 140)	*P*-values
Age (years)			< 0.001
40–59	268 (39.07%)	24 (17.14%)	
60–74	319 (46.50%)	54 (38.57%)	
≥ 75	99 (14.43%)	62 (44.29%)	
BMI (kg/m^2^)			< 0.001
< 18.5	19 (2.77%)	35 (25%)	
18.5–24	302 (44.02%)	81 (57.86%)	
> 24	365 (53.21%)	24 (17.14%)	
Hypertension			0.358
Yes	141 (20.55%)	24 (17.14%)	
No	545 (79.45%)	116 (82.86%)	
Diabetes mellitus			0.089
Yes	71 (10.35%)	8 (5.71%)	
No	615 (89.65%)	132 (94.29%)	
Fracture history			< 0.001
Yes	56 (8.16%)	36 (25.71%)	
No	630 (91.84%)	104 (74.29%)	
Cardiovascular disease			< 0.001
Yes	71 (11.54%)	30 (21.43%)	
No	615 (89.65%)	110 (78.57%)	
Cerebrovascular disease			< 0.001
Yes	29 (4.23%)	20 (14.29%)	
No	657 (95.77%)	120 (85.71%)	
Tumor			0.458
Yes	21 (3.06%)	6 (4.29%)	
No	665 (96.94%)	134 (95.71%)	
CRF			0.216
Yes	13 (1.90%)	5 (3.57%)	
No	673 (98.10%)	135 (96.43%)	
Thyroid disease			0.042
Yes	11 (1.60%)	6 (4.29%)	
No	675 (98.40%)	134 (95.71%)	
Rheumatism			1.000
Yes	5 (0.73%)	1 (0.71%)	
No	681 (99.27%)	139 (99.29%)	
Neuropathy			0.148
Yes	22 (3.21%)	8 (5.71%)	
No	664 (96.79%)	132 (94.29%)	
ESR (mm/h)			< 0.001
≤ 20	600 (87.46%)	103 (73.57%)	
> 20	86 (12.54%)	37 (26.43%)	
WBC (10^9^/L)			< 0.001
< 4	17 (2.48%)	6 (4.29%)	
4–10	617 (89.94%)	110 (78.57%)	
> 10	38 (5.54%)	24 (17.14%)	
CRP (mg/L)			< 0.001
≤ 8	567 (82.65%)	93 (66.43%)	
> 8	119 (17.35%)	47 (33.57%)	

BMI, body mass index; CRF: chronic renal failure; CRP, C-reactive protein; ESR, erythrocyte sedimentation rate; WBC, white blood cell count. *P* < 0.05 (two-sided) was considered statistically significant.

### Cut-off value of systemic inflammatory markers

**Figure 1:**
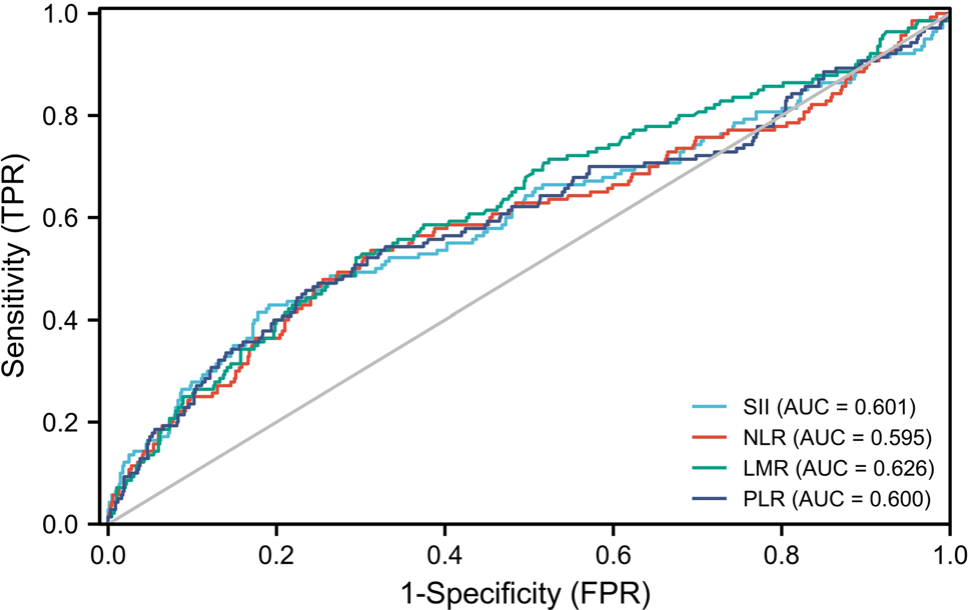
Receiver operating characteristic curve of a systemic immune-inflammation index (SII), neutrophil-to-lymphocyte ratio (NLR), lymphocyte-to-monocyte ratio (LMR), and platelet-to-lymphocyte ratio (PLR). The cut-off value for SII, NLR, LMR and PLR were 725.00 (area under the curve, 0.601; P < 0.001), 2.52 (area under the curve, 0.595; P < 0.001), 3.23 (area under the curve, 0.626; P < 0.001), 147.80 (area under the curve, 0.600; P < 0.001), respectively.

The appropriate cut-off value for SII, NLR, LMR, and PLR were 725.00 (area under the curve, 0.601; *P* < 0.001, 95%CI 0.541–0.657), 2.52 (area under the curve, 0.595; *P* < 0.001, 95%CI 0.536–0.653), 3.23 (area under the curve, 0.626; *P* < 0.001, 95%CI 0.593–0.659), 147.80 (area under the curve, 0.600; *P* < 0.001, 95%CI 0.546–0.656), respectively (Fig. 1). As shown in Table 2, the systemic inflammatory markers (SII, NLR, LMR, and PLR) were higher in the OP group than in the non-OP group (all *P* value < 0.001).

**Table 2:** Comparison of the systemic inflammatory markers of male patients. Categorical parameters were shown as percentage.

Parameters	Non-OP (n = 686)	OP (n = 140)	*P*-values
SII			< 0.001
≤ 725.00	555 (80.90%)	80 (57.14%)	
> 725.00	131 (19.10%)	60 (42.86%)	
NLR			< 0.001
≤ 2.52	472 (68.80%)	66 (47.14%)	
> 2.52	214 (31.20%)	74 (52.86%)	
LMR			< 0.001
≤ 3.23	202 (29.45%)	73 (52.14%)	
> 3.23	484 (70.55%)	67 (47.86%)	
PLR			< 0.001
≤ 147.80	525 (76.53%)	76 (54.29%)	
> 147.80	161 (23.47%)	64 (45.71%)	

LMR, lymphocyte-to-monocyte ratio; NLR, neutrophil-to-lymphocyte ratio; PLR, platelet-to-lymphocyte ratio; SII, systemic immune-inflammation index. *P* < 0.05 (two-sided) was considered statistically significant.

### Feature selection

**Figure 2:**
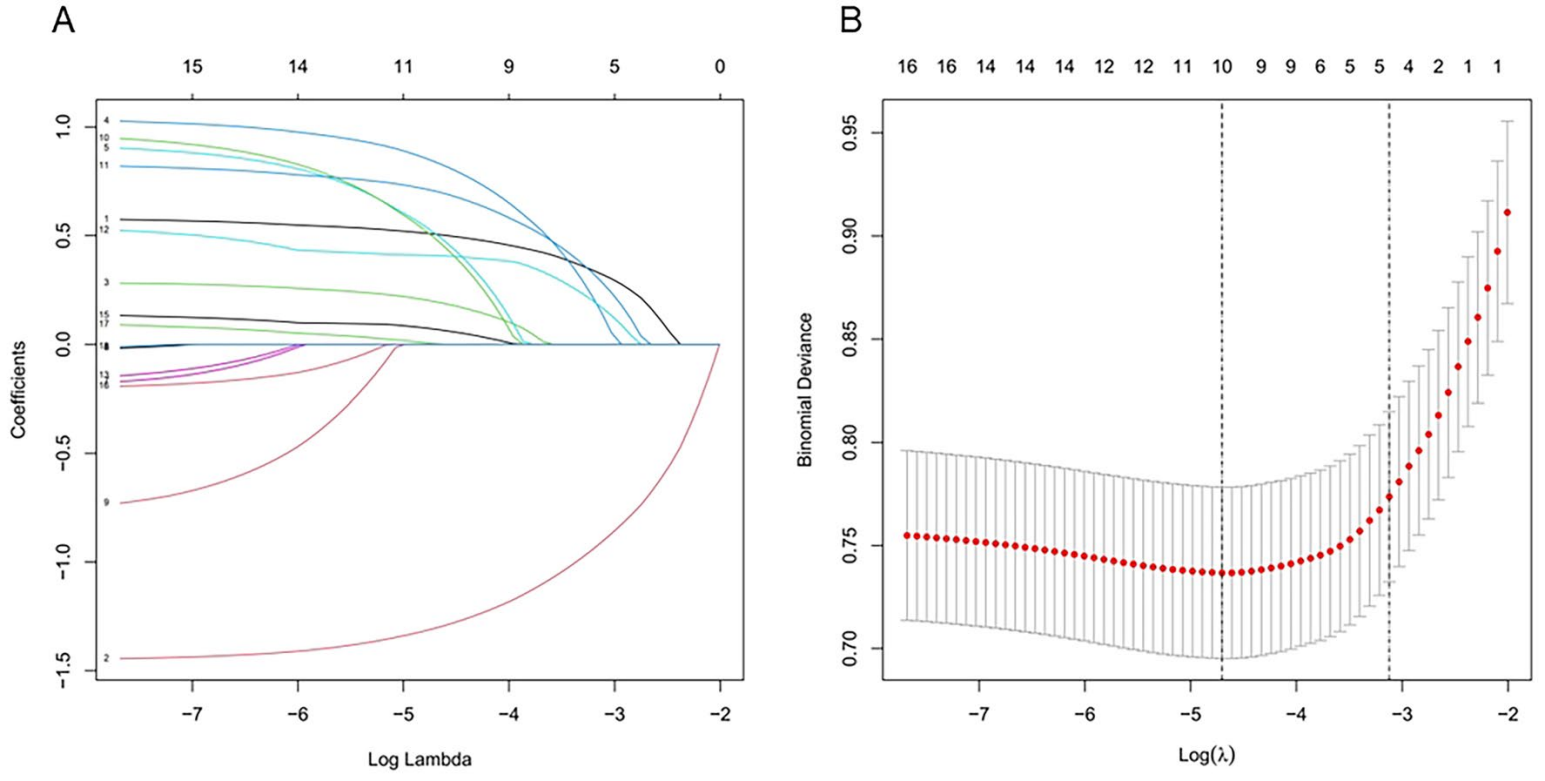
Feature selection using the LASSO binary logistic regression model. (**A**) LASSO coefficient profiles of 19 features. A coefficient profile picture was generated against the log (lambda) sequences. Vertical line was drawn at the value selected using fivefold cross-validation, where optimal lambda resulted in 10 features with nonzero coefficients. (**B**) Optimal lambda selection in the LASSO model used fivefold cross-validation via minimum criteria. The partial likelihood deviance curve was plotted versus lambda. Dotted vertical lines were drawn at the optimal values by using Lambda.min of 0.00002188 and Lambda.1se of 0.00066069.

The variables in present study included demographic, disease, and blood biochemical characteristic. We selected 10 characteristic variables from 19 variables through LASSO regression model with nonzero coefficients (Fig. 2A and B). These variables included age, body mass index (BMI), cardiovascular diseases, cerebrovascular disease, neuropathy, thyroid disease, fracture history, SII, PLR, CRP (Table 3).

**Table 3:** Prediction factors for osteoporosis in Chinese male patients.

Parameters	β	Wald	P	OR	95%CI
Intercept	− 0.585	− 1.551	0.121	0.557	0.264–1.168
Age (years)					
40–59					Reference
60–74	0.416	1.480	0.139	1.516	0.882–2.668
≥ 75	1.118	3.565	< 0.001	3.058	1.664–5.711
BMI (kg/m^2^)					
< 18.5					Reference
18.5–24	− 1.762	− 5.192	< 0.001	0.172	0.087–0.331
> 24	− 2.995	− 7.823	< 0.001	0.050	0.023–0.104
Cardiovascular disease	0.295	1.019	0.308	1.344	0.752–2.348
Cerebrovascular disease	1.053	2.907	0.004	2.865	1.392–5.794
Neuropathy	0.821	1.654	0.098	2.273	0.818–5.830
Thyroid disease	0.803	1.219	0.223	2.231	0.581–7.818
Fracture history	0.829	2.724	0.006	2.292	1.255–4.151
SII					
≤ 725					Reference
> 725	0.402	1.298	0.194	1.495	0.811–2.741
PLR					
≤ 147.8					Reference
> 147.8	0.156	0.534	0.594	1.168	0.653–2.054
CRP					
≤ 8					Reference
> 8	0.041	0.144	0.886	1.041	0.592–1.796

BMI, body mass index; CRP, C-reactive protein; PLR, platelet-to-lymphocyte ratio; SII, systemic immune-inflammation index. *β* is the regression coefficient. *P* < 0.05 (two-sided) was considered statistically significant.

### Development of an individualized prediction model

**Figure 3:**
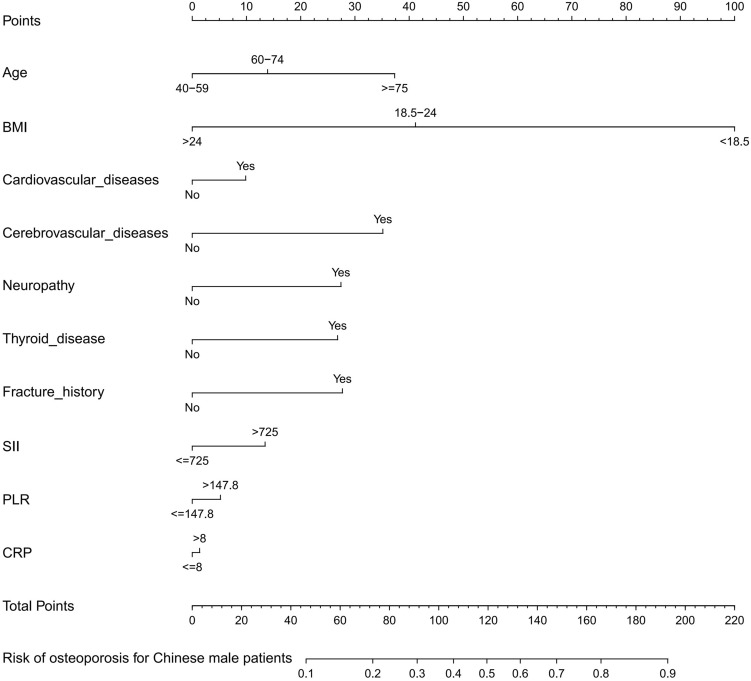
Developed osteoporosis risk nomogram. The nomogram was built with cohort, with age, BMI, cardiovascular disease, cerebrovascular disease, neuropathy, thyroid disease, fracture history, SII, PLR, CRP incorporated.

We utilized all ten feature variables chosen from the LASSO regression model to construct the prediction model for the clinical practicality, following a selection method similar to previous literature ^20,21^. The predictive model was construct using multiple logistic regression method (Table 3), and the predictive model constructed by the predictors mentioned above was displayed as a nomogram (Fig. 3).

### Apparent performance of the OP risk nomogram in the cohort

**Figure 4:**
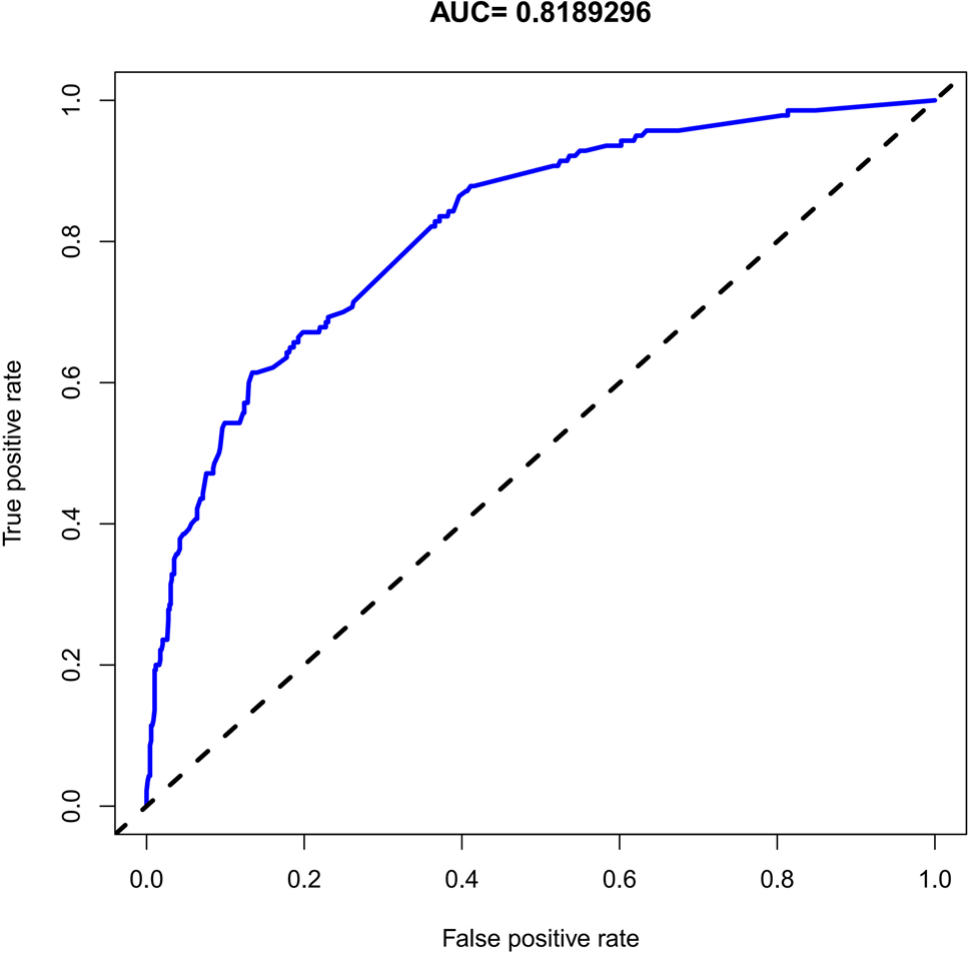
Receiver operating characteristic curve of the predictive OP risk nomogram. The Y-axis represents the TPR of the risk prediction, the X-axis represents the FPR of the risk prediction. The blue line represents the performance of the nomogram.

**Figure 5:**
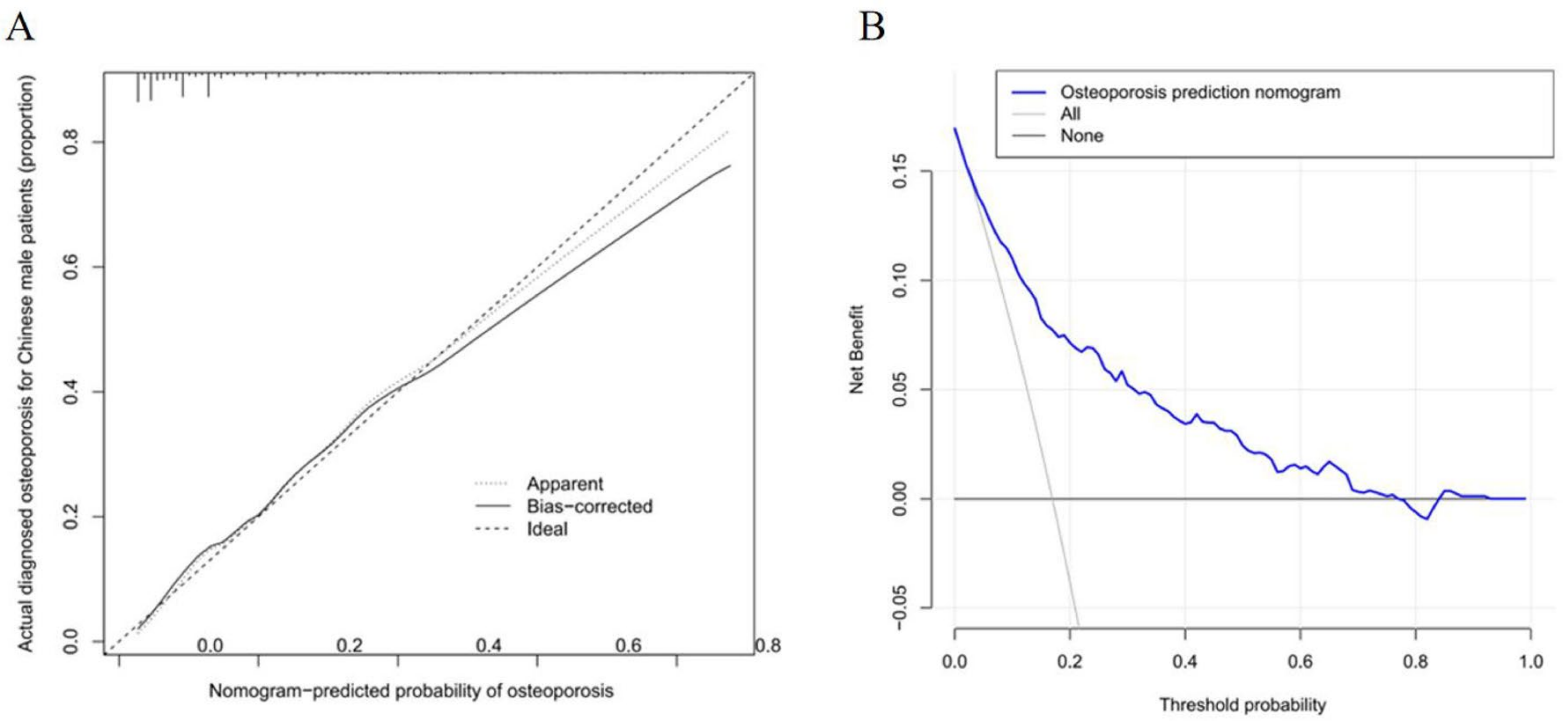
(**A**) Calibration curves of the OP risk nomogram prediction in the cohort. The X-axis represents the predicted osteoporosis risk. The Y-axis represents the actual diagnosed OP. The dotted line represents the OP risk nomogram. The thin solid line represents the assumption that all patients are subjected of OP. (**B**) Decision curve analysis of the OP risk nomogram. The Y-axis measures the net benefit.

The area under the ROC (AUC) of the risk nomogram was 0.822 (CI 0.798–0.846) (Fig. 4), which suggested that the model had adequate predictive potential and performance. The calibration curve of the OP risk nomogram for the prediction of OP risk in male based on features such as SII and PLR illustrated good agreement in this cohort (Fig. 5A). The *C*-index for this nomogram was 0.822 (CI 0.798–0.846) for the cohort, and was confirm to be 0.8048 through bootstrapping validation, which suggested this prediction model had a good discrimination. In the OP risk nomogram, apparent performance addressed a good prediction capability.

### Clinical use

The decision curve analysis (DCA) for the male OP risk nomogram is shown in Fig. 5B. The DCA curve showed that using the risk nomogram to predict the risk of OP in the male population is beneficial in clinical practice when the threshold probability of OP is between 3 and 76%. Within this range, net benefit was comparable with several overlaps.

## Discussion

Bone formation and metabolism are regulated by the immune system of the body, where key mechanisms may involve age-increasing oxidative stress and low-level activation of immune system. Measurement of the number of immune cells present in the blood can be used as an indicator of systemic inflammation, with correspondence to changes in bone metabolism state. These immune cells count, commonly found during blood during routine blood examinations, help provide better understanding of the osteopathology^22^. Immune cells such as lymphocytes can modulate the immune microenvironment by secreting cytokines, thereby influencing bone homeostasis. For example, T lymphocytes and other inflammation-related cells produce large amounts of cytokines that stimulate the RANKL/RANK/OPG pathway, promoting osteoclastogenesis and thus resulting in imbalance of bone homeostasis^23^. B lymphocytes can secrete OCP to compete with RANKL to inhibit osteoclasts and thus slow down bone resorption; however, B cells can also express RANKL in response to inflammatory stimuli, further augmenting osteoclastogenesis. When lymphocytes become dysfunctional, a cascade of inflammatory factors and chemokines may be triggered, resulting in accumulation of neutrophils and macrophages and thus leading to an imbalance in bone homeostasis and increased bone resorption^24^. Neutrophils can express OPG and RANK into the cell membrane when stimulated by IL-4 or TNF-α, for instance^25^. Furthermore, they can release precursors such as CCL2 and CCL20 to recruit Th17 cells, which can ultimately result in bone loss^26^. Platelets are fragments of cytoplasm derived from the megakaryocytes of the bone marrow, which also play an important role in bone homeostasis^27^. Previous clinical study^28^ has found that PLR was negatively correlated with BMD in postmenopausal OP patients, which was also related to low vitamin D levels, suggesting that inflammation correlates with vitamin D either. In addition, several vitro studies showed that platelet contributed to bone resorption^29^. However, the specific mechanism is uncertain due to the complicated interaction between grow factors, inflammatory mediators and cytokines.

The recently reported systemic inflammatory markers like SII, NLR, LMR and PLR^30^ are calculated by immune cell count in blood and have demonstrated strong diagnostic and predictive capabilities in infectious, cancer and autoimmune diseases^31,32^. Fang et al.^11^ demonstrated that systemic inflammatory markers such as SII, NLR and PLR were higher in postmenopausal women than in premenopausal women. These markers also demonstrated good predictive value for osteoporotic fracture in postmenopausal women. However, the relationship between systemic inflammatory markers and the incidence of OP in men is still unclear, and current assessment tools are inadequate. To develop a targeted and personalized assessment tool, we constructed a novel nomogram for evaluate the risk of OP in Chinese male population based on demographic data and systemic inflammatory markers. In our study, we found that age, BMI, cardiovascular disease, cerebrovascular disease, neuropathy, thyroid disease, fracture history, SII, and PLR possibly contributed to the risk of OP in men. The systemic immune-inflammation indexes included in a large cross-sectional study of bone mineral density in postmenopausal women by Tang et al. includes SII, NLR, PLR and PPN(the product of platelet count and neutrophil count)^33^. However, we did not include PPN in this study because there is no authoritative verification at present. The remaining three special inflammatory markers are SII, NLR and PLR, whose mechanisms of action has been verified to have a good predictive value for OP and fracture risk due to the disruption of bone homeostasis caused by the activation of the inflammatory microenvironment and a compromised immune system^34^. Similarly, for the LMR biomarkers included in this study, there are also related literature reports that this inflammatory marker is an indicator of the immune response status and overall inflammation level ^35^, which is consistent with the mechanism of bone metabolism.

In order to screen appropriate variables from multiple independent factors, the LASSO regression model was conducted in this study. The LASSO regression model is a classic type of machine learning model, which is a penalized regression of all variable coefficient so that relatively insignificant independent variable coefficients become zero and are thus excluded from the modeling^36^. In addition, LASSO regression allows for variable screening and complexity adjustment while fitting a generalized linear model. The results of our study showed that the model constructed using the variables selected by LASSO regression has relatively good accuracy and predictive ability.

In this study, subsequent to the exclusion of alternative systemic inflammatory markers, the LASSO regression model ultimately selected SII and PLR. Although these variables did not achieve statistical within the multiple logistic regression analysis, the importance of variables SII and PLR in the diagnosis of OP should not be diminished. Both markers are acknowledged to represent the state of immune-related inflammation to a certain degree^37^, so they can provide valuable insight into the patient’s overall health condition. Thus, these two indices were finally included in the nomogram. Since SII and PLR can be feasibly accessed from routine blood indicators, this nomogram can be practical and convenient for physicians to use.

In our present study, 20.41% of patients were diagnosed with OP. In the risk factor analysis, age, BMI, cerebrovascular disease, and fracture history were associated with the OP. First, as known to all, ageing is no doubt a certain risk factor for OP^38^. The main causes of age-related bone loss are sex steroid production, including sex hormone and age-related hypogonadism^39^. As for men population, the sustained loss of testosterone could accelerate bone loss^40^. On the other hand, it has been demonstrated that the lower oestrogen levels, the higher the bone loss^41^. In addition, Yousefzadeh et al.^42^ illustrated that an aged, senescent immune system could drive systemic ageing of solid organism. While this study did not focus on the pathology of the OP, which indicated that aged immune system would affect skeletal system. Second, lower body weight is a risk factor for acquiring OP^43^, and obesity is considered to act as a protective effect on bone. This is consistent with our findings. Although the exact mechanism has been elucidated, increased aromatization of estrogen to androgen in adipose tissue is accompanied by decreased concentrations of sex hormone-binding globulin and hyperinsulinemia, which may be a mitotic factor for osteoblast^44^. Third, according to recent researches, cerebrovascular disease generally considered to be associated with OP^45^. Zhang et al.^46^ conducted a study on the database of the Taiwan population, and concluded that patients with stroke have a higher risk of OP, and post stroke OP was significant in male gender, which is also consistent to our study. Previous study^47^ used OP related factors to predict risk of fracture in Chinese male population. Interestingly, our study found that a history of fracture is also an important factor in aiding the diagnosis of OP with male gender before a definitive diagnosis is made.

## Limitation

There are also several limitations of our current study. First, in the multivariate logistic regression analysis, SII and PLR were not statistically significant. Nonetheless, they were screened by LASSO regression and eventually included in the model due to their own clinical relevance. Second, the cohort was collected over a period of time in a single center, so the included male patients were not representative of all male population in Chinese area. Third, although we validated the model internally by using bootstrap testing, the lack of sample size caused the absence of external validation. Therefore, future study will perform external validation by expanding the sample size and optimize the calibration of predictive models to demonstrate the robustness of the model. Additionally, enhancing the model's calibration will be crucial to improve its clinical utility, as proper calibration ensures the model's predicted probabilities of disease align closely with actual outcomes.

## Conclusion

OP with male patients is associated with increased level of systemic inflammatory markers. In this study, we successfully developed a novel nomogram based on SII and PLR to accurately predict OP in male population. If further study through external verification, this may be an accessible tool for assisting clinicians rapidly screening for male who are susceptible to OP.

## Data Availability

The datasets generated and/or analysed during the current study are not publicly available due ethical reasons but are available from the corresponding author on reasonable request.
